# Glow discharge plasma stabilization of azo dye on PMMA polymer

**DOI:** 10.1038/s41598-022-21855-4

**Published:** 2022-11-01

**Authors:** Mohammad Reza Yari, Mohammad Sadegh Zakerhamidi, Hamid Ghomi

**Affiliations:** 1 Laser and Plasma Research Institute, Shahid Beheshti University, Tehran, Iran; 2 Faculty of Physics, University of Tabriz, Tabriz, Iran; 3 Research Institute for Applied Physics and Astronomy, University of Tabriz, Tabriz, Iran; 4 Photonics Center of Excellence, University of Tabriz, Tabriz, Iran

**Keywords:** Plasma physics, Electronics, photonics and device physics, Photonic devices, Polymer chemistry, Surface chemistry

## Abstract

The effects of argon gas glow discharge plasma on the surface of DR1 dye-loaded PMMA polymer films are examined in this work. Plasma immobilizes the dye on the surface of polymer without using stabilizers. Argon plasma activates the surface through breaking some bonds and generation of radical sites. It affects the acrylate groups of PMMA leading to covalent bonds between dye and surface of polymer. In addition, plasma treatment and contact with ambient air may result in the creation of new polar components, such as carbonyl and carboxyl compounds and links that enhance the dye attachment to the polymer matrix. Besides, the dye adsorption on the polymer film is impacted by changes in surface topography. Furthermore, plasma modifies the dye conformation, which affects the adherence of the dye to the polymer surface through bringing the dye to the higher energy state. The chemical and topographical modification of dye-loaded PMMA films by plasma are investigated by spectroscopic and AFM methods. Furthermore, aging process was used to confirm dye retention on the polymer film after plasma modification as opposed to dye-loaded polymer film that was left untreated as a reference sample. Finally, investigated method suggests a novel and very affordable technique for fabrication of poly(MMA-co-DR1) copolymer in the form of a homogeneous surface layer.

## Introduction

Environmental stability, proper optical characteristics, low cost^1^, chemical inertness, high flexibility^2^, and mechanical strength superior to glass^3^ are all properties of poly methyl methacrylate (PMMA). PMMA is widely used in a variety of industries, such as biomedical devices^4^, sensors^5^, and optoelectronics^6^. This polymer is a low-density transparent and hydrophobic thermoplastic^2^. It also has a low surface free energy which results in poor adhesion^7^.

Surface characteristics are critical for a variety of polymer applications. Changing surface properties, such as adhesion^8^, printing, and biocompatibility^9^, which are defined in the surface of material, expands the range of applications of polymers. In general, polymers are modified to improve physical and chemical properties, for example, creating suitable superficial functional groups, change in polarity^10^, wettability, roughness^11^, enhancement of surface free energy^12^, reflection variation^6^, surface etching, creating nanotextured surfaces^13^, and changing adhesion properties^14^. There are various techniques to modify the surface of polymer, such as chemical modification^15^, thermal treatment^16^, flame treatment^17^, ion beam treatment^18^, and radiation^19^. The use of different harmful chemicals, costly equipment, dangerous radiation, and high energy consumption are the difficulties of utilization of these methods.

Plasma surface modification of materials is a clean, efficient, rapid, homogeneous, and environmentally friendly process^20^. It works with a variety of materials, including metals^21^, composites^22^, and polymers^23^. This technique can change surface properties without affecting the favorable bulk properties of material^24^. Surface modification of polymers using non-thermal plasma leads to surface activation and produces new functional groups^25^. Plasma impresses the surface of polymers with thicknesses ranging from nanometers to micrometers^8^. Plasmas contain highly reactive, excited, and energetic particles, such as electrons, radicals, metastable species, and ultraviolet radiation, which interact with the surface of material^26^. Some patterns are formed on polymeric surfaces during plasma treatment, and energetic particles can influence some structural bonds of polymers, resulting in phenomena such as cross-linking, degradation, recombination, etching, oxygenation, and depolymerization^27,28^. Also, the surface energy of the polymer can be increased by plasma^29^. Furthermore, surface charging of polymers is an unavoidable plasma effect^30^. As a result, plasma can change some surface properties of polymers like wettability, biocompatibility, printability, roughness, and adhesion^31,32^. The properties of plasma-treated polymer surfaces are influenced by a number of plasma parameters, including power density, treatment time, gas flow rate, pressure, gas type, reactor configuration, and sample location^33,34^, which change some variables, such as electron density, mean electron energy, and the number of radicals. The low surface energy and hydrophobic properties of PMMA polymer limit its applications and reduce surface adhesion^35^. These variables can be influenced simply by modification of a monomolecular layer^36^. Sputtering, ionization, excitation, breaking some bonds, and introduction of new species occur during plasma surface modification of PMMA, resulting in chemical and physical changes to the polymer's surface^37^. Additionally, some polar functional groups and reactive species are generated on the surface, which improve adhesion, surface energy, and hydrophilicity^38^. Furthermore, the creation of a nano-pattern surface improves the surface adhesion of PMMA polymer^39^.

Azobenzenes as molecular switching systems have two space conformations called trans (E) and cis (Z) isomers, which trans state is converted to cis mode under the irradiation of ultraviolet rays^40^. Photoisomerization of azobenzene from trans to cis state changes the spatial shape of the azobenzene molecule by closing the aromatic rings together^41^. The cis isomer has a higher energy state than the trans isomer (about 50 kJ/mol), and the cis mode can be converted to the trans mode by losing energy and thermal relaxation^42^. Azobenzenes are used in various cases, such as sensors^43^, data storage^44^, nonlinear optics^45^, nano machines^46^, drug delivery^47^, holographic works^48^. Disperse Red 1 (DR1) dye, as one of the most well-known dyes of this group of the dyes, also has halochromic properties that can be used to determine PH changes when the color is changed^49^. As a result, it is critical to keep DR1 dye stable on the surface for various applications. Surface morphology, hydrophilicity, and surface chemistry, as well as charge and energy, all play a role in dye immobilization on the surface of polymer^50^. There are several methods for immobilizing dye on the surface such as adding a polymeric fixating agent, but none of them completely prevent dye fading^51^.

In this study, glow discharge plasma with argon gas was applied to treat pure and dye-loaded PMMA polymer films to investigate the effects of plasma on the surface. In order to determine chemical and topographical changes of the surface of PMMA films induced by plasma, Fourier-transform infrared spectroscopy (FT-IR) and atomic force microscopy (AFM) were used. The durability of dye on the surface of polymer after plasma treatment was investigated using an accelerated aging process. UV–Vis spectroscopy was used to determine dye cis–trans isomer composition changes before and after plasma, as well as the results of the aging process. There has been an attempt to propose reliable mechanisms for the effects of plasma on dye-loaded polymers.

## Results

### Plasma effects on pure and DR1 doped PMMA polymer films

**Figure 1:**
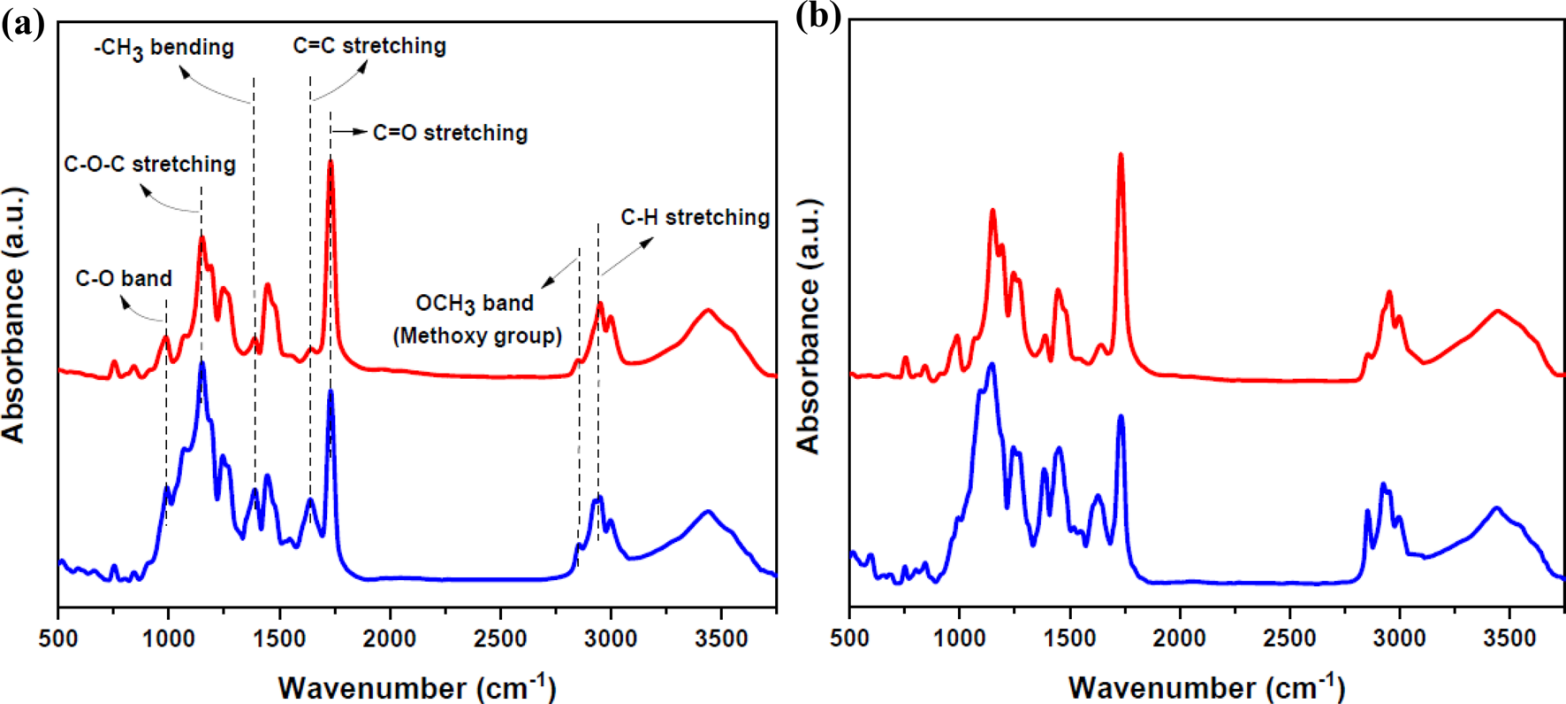
FT-IR spectra of (**a**) pure PMMA polymer layer (**b**) DR1 dye-doped PMMA polymer layer and (blue lines) untreated samples (red lines) argon plasma-treated samples for 300 s modification.

The FT-IR spectra of both pure and DR1 dye-doped PMMA thin films were studied before and after the argon plasma treatment for 300 s. This optimized time interval of treatment of plasma with known characteristics in this work, was obtained by trial and error. FT-IR analysis revealed the chemical and structural changes caused by plasma treatment in both pure and dye-doped polymers. The FT-IR spectra of pristine and plasma-treated films are shown in Fig. 1. Also, Supplementary Fig. S1 in the supplementary information presents more detailed peaks information of FT-IR spectra of Fig. 1a. And for a better comparison, Supplementary Fig. S2 in the supplementary information indicates the spectra of pure and DR1 dye-doped PMMA polymer before and after plasma treatment for 150 s.

The FT-IR spectra of the samples demonstrate the absorption bands in the region of 2920–3000 cm^−1^ attributed to C–H symmetric and asymmetric stretching vibrations. The peak at 2850 cm^−1^ is related to methoxy group OCH_3_ in the side group of PMMA backbone. The characteristic intense absorbing band appearing at 1732 cm^−1^ is assigned to carbonyl group C=O stretching. On the other hand, the observed band at 1647 cm^−1^ corresponds to C=C bond stretching vibration. The dual peaks at 1485 and 1450 cm^−1^ are ascribed to the asymmetric bending modes of the –CH_2_ and –CH_3_ groups. Also, the peak at 1388 cm^−1^ is due to −CH_3_ bending vibration. The peaks located at 1271 and 1243 cm^−1^ are interpreted as C–C–O stretching vibration band. The typical absorption bands are observed at 1193 and 1149 cm^−1^ originate from C–O–C stretching vibration. The peaks in the range of 990–1090 cm^−1^ refer to stretching C–O bonds in PMMA structure.

**Figure 2:**
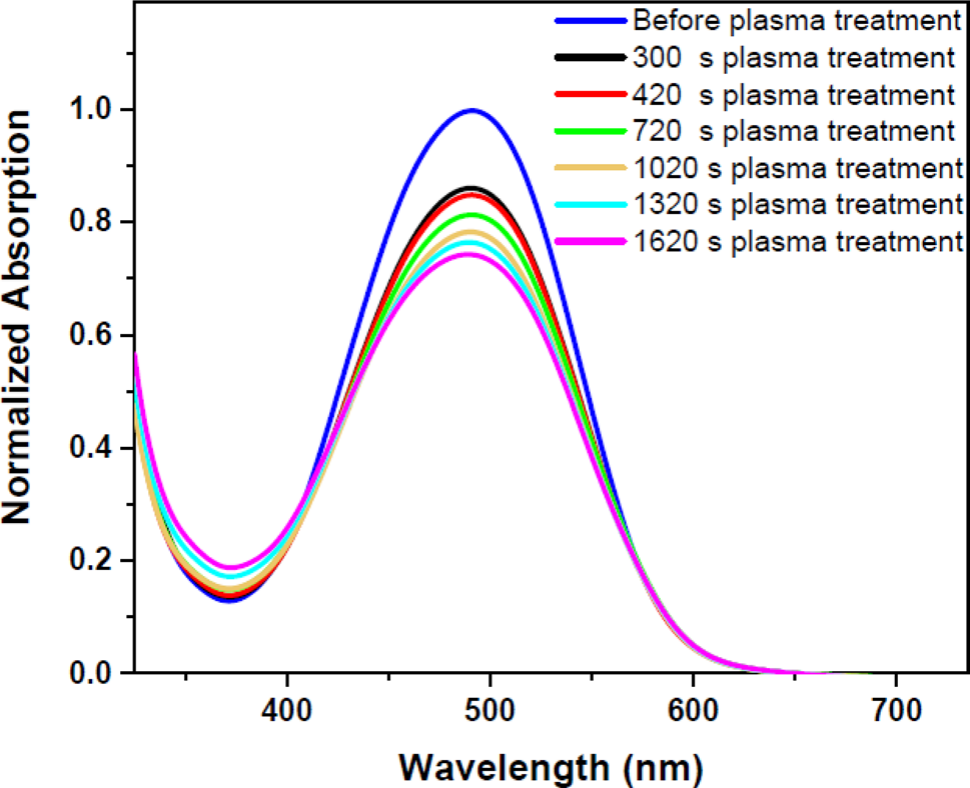
UV–Vis spectra of pristine and argon plasma-treated DR1 dye-doped PMMA polymer film for various treatment times.

**Figure 3:**
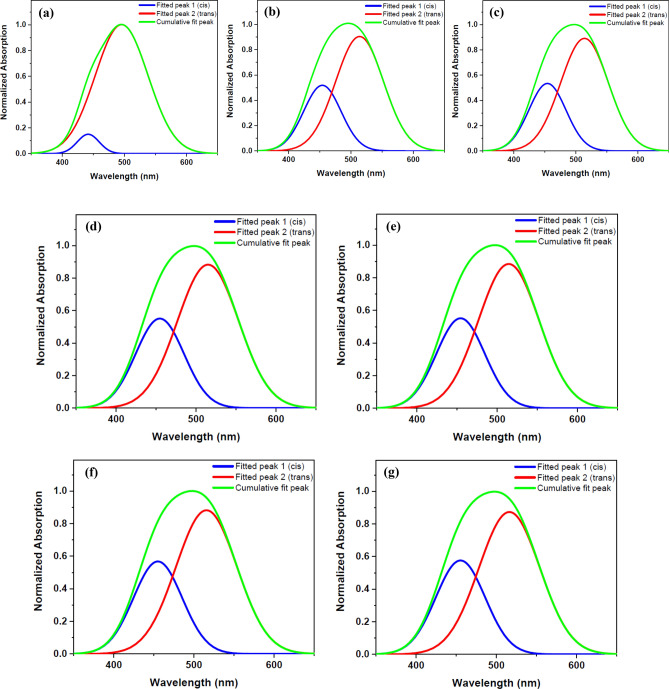
Double peaks fitted for the UV–Vis normalized absorption spectra of DR1 dye-doped PMMA polymer film before and after plasma modification for different treatment times: (**a**) before plasma treatment (**b**) 300 s (**c**) 420 s (**d**) 720 s (**e**) 1020 s (**f**) 1320 s (**g**) 1620 s plasma treatment.

The DR1 dye-doped PMMA polymer film was exposed to plasma for various times to investigate other plasma-induced changes which is seen in Fig. 2. In this part of experiment, the effects of argon glow discharge plasma on the sample at different times were compared by UV–Vis spectra. Figure 2 shows the UV–Vis spectra of the sample before and after plasma treatment at various time intervals. Figure 3 shows the deconvoluted peaks of each spectrum of Fig. 2 that indicates the overlapped peaks related to cis and trans forms of the dye. Table 1 presents the area under each fitted peak related to the population of cis and trans isomers of the dye.

**Table 1:** The amounts of DR1 dye isomeric species and their ratios calculated from fitted double peaks before and after plasma treatment at different time intervals for dye-doped PMMA.

Samples	S_trans_	S_cis_	S_cis_/S_trans_	S_cis_/S_total_(%)
Before plasma treatment	118.44	7.37	0.06	7.69
300 s plasma treatment	92.06	21.18	0.23	18.70
420 s plasma treatment	91.83	21.61	0.23	19.04
720 s plasma treatment	89.80	23.85	0.25	20.98
1020 s plasma treatment	87.57	24.52	0.28	21.87
1320 s plasma treatment	80.18	24.12	0.30	23.12
1620 s plasma treatment	79.57	25.20	0.31	24.05

*S*_*trans*_ area under the fitted peak 1, *S*_*cis*_ area under the right fitted peak 2, *S*_*total*_ area under the cumulative fit peak.

**Figure 4:**
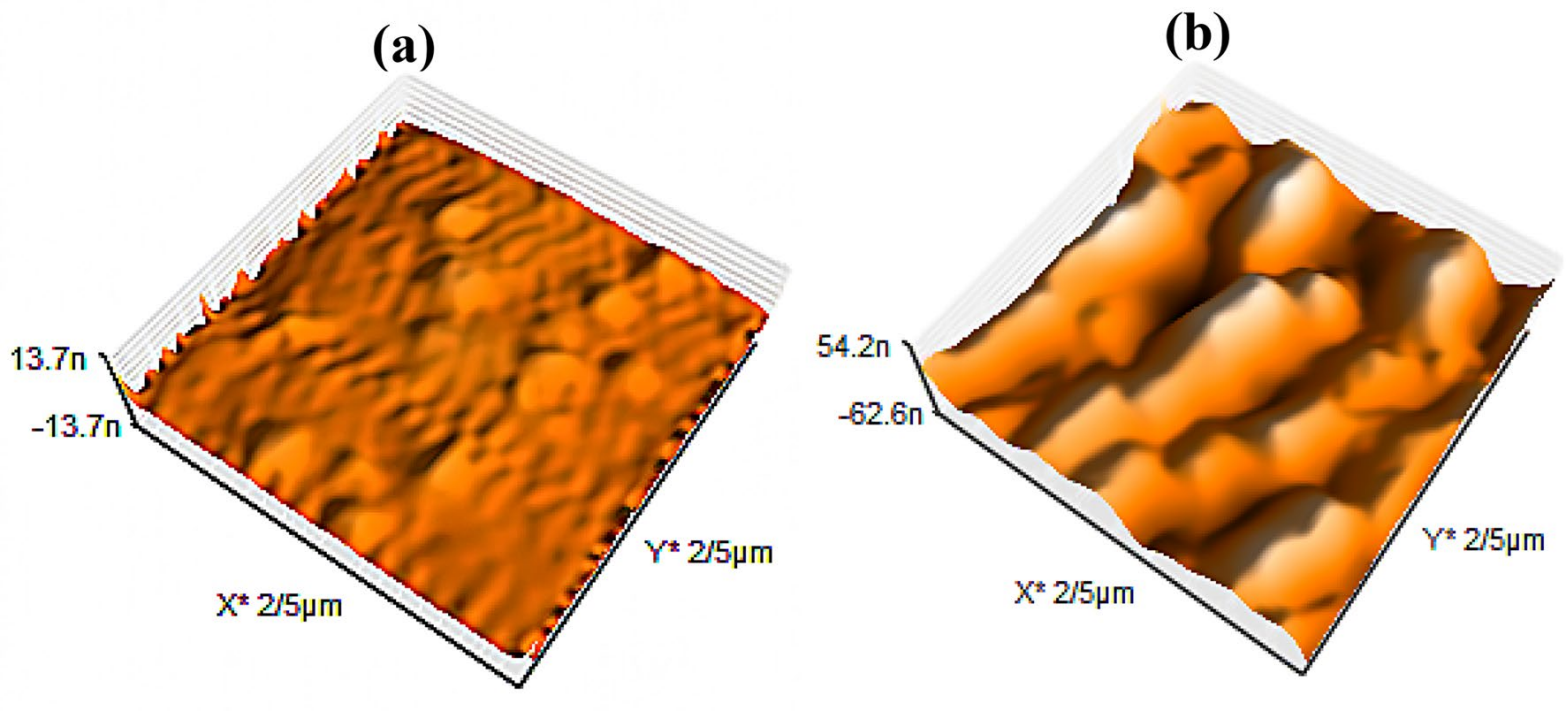
AFM images of (**a**) untreated (**b**) argon plasma-treated DR1 dye-doped PMMA surface for 300 s modification.

Given the effects of plasma on the surface, using surface topographical images to examine the changes on the surface is a viable approach. The data produced from 3D topographical AFM images is quite useful in identifying material surface structure. AFM microscope was used to examine the surface topography of DR1 dye-doped PMMA films. AFM images of pristine and 300 s argon plasma-treated samples are shown in Fig. 4. Also, Supplementary Fig. S3 in supplementary information shows surface roughness before and after plasma treatment for 150 s.

As demonstrated in Fig. 4, argon plasma treatment for 300 s significantly affected the surface topography and roughness of DR1 dye-doped PMMA. Table 2 shows some of the surface roughness parameters before and after argon plasma treatment.

**Table 2:** Comparative presentation of roughness parameters (R_a_, R_q_, and R_z_) of dye-doped PMMA polymer surfaces before and after argon glow discharge plasma treatment for 300 s.

Samples	Roughness parameters		
	R_a_ (nm)	R_q_ (nm)	R_z_ (nm)
Untreated DR1 dye doped PMMA	0.838	1.023	5.107
plasma-treated DR1 dye doped PMMA	9.205	11.306	51.455

The roughness parameters include the arithmetic averages of the assessed values (R_a_), the root mean square average of height deviations from the mean line (R_q_), and the maximum peak to valley height (R_z_).

### Plasma effect on surface dyeing of DR1 dye-doped PMMA

**Figure 5:**
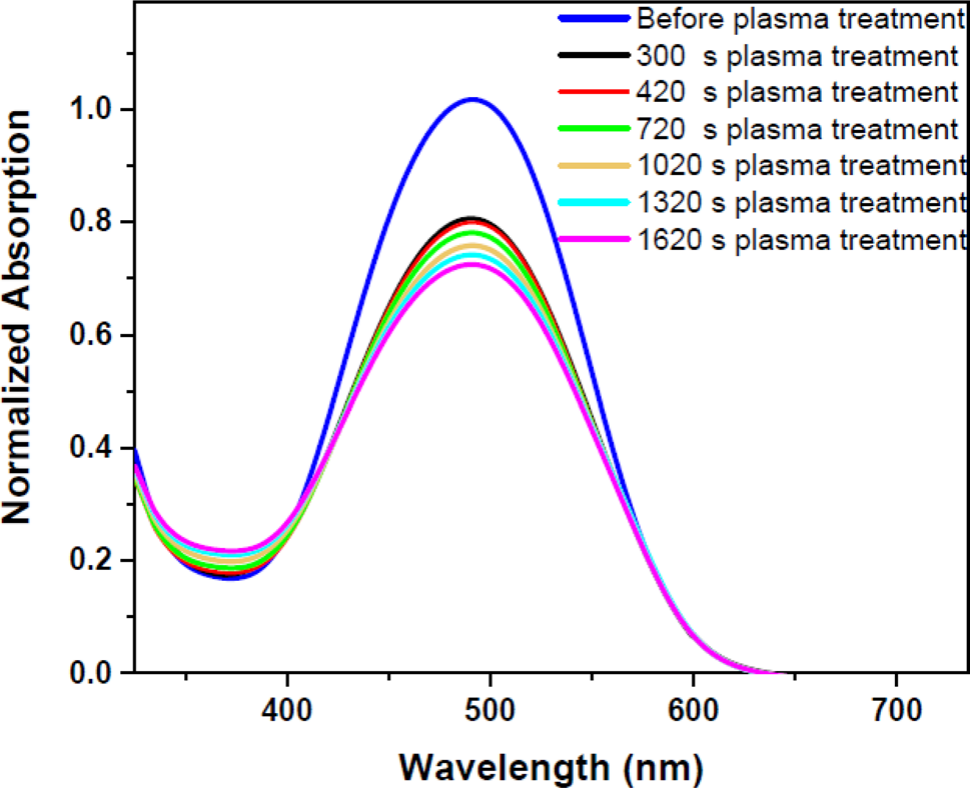
UV–Vis spectra of pristine and DR1 dye-doped PMMA polymer film with extra adsorbed surface dye through immersing in DR1 dye solution treated with argon plasma at various treatment times.

**Figure 6:**
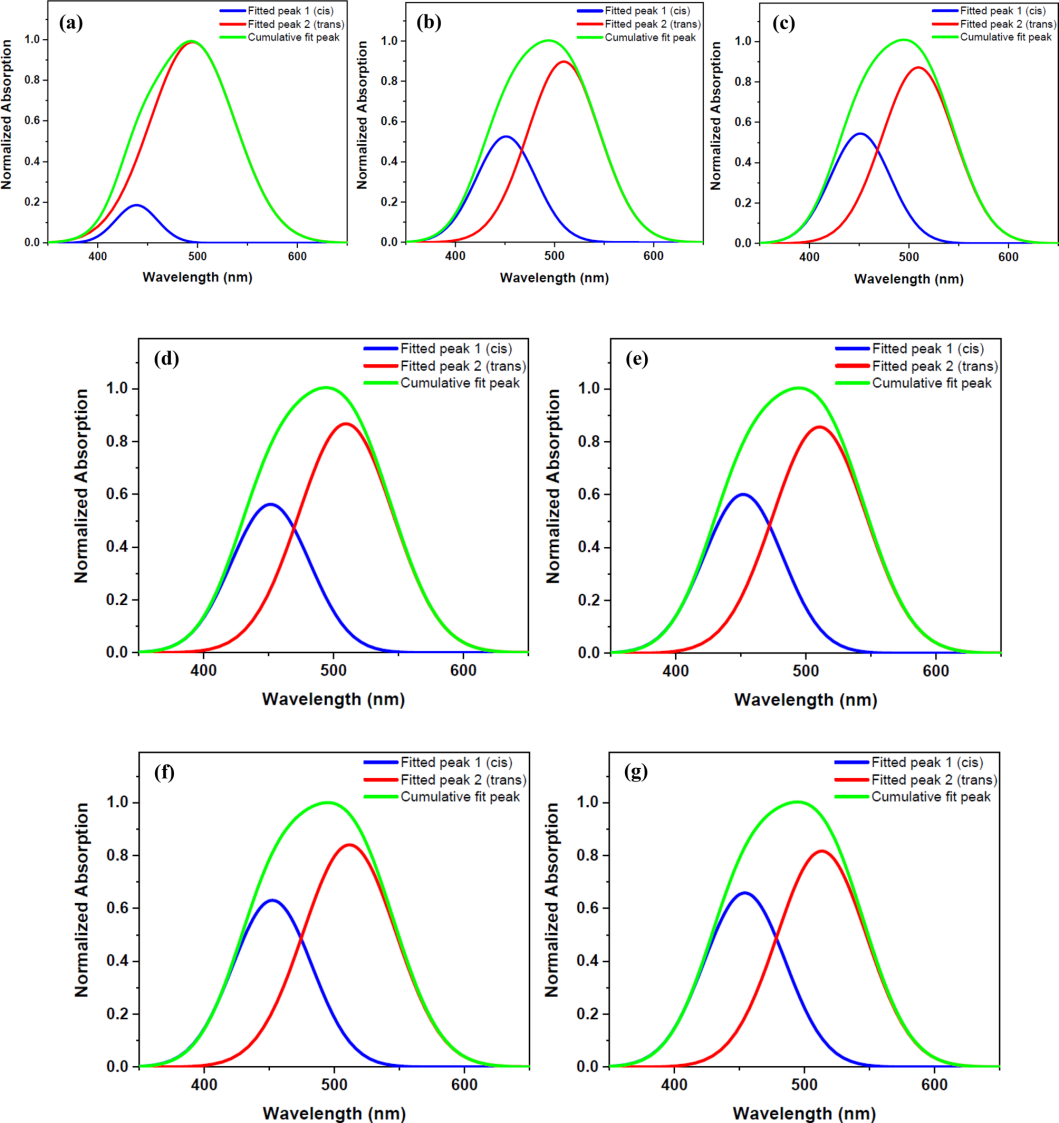
Double peaks fitted for the UV–Vis normalized absorption spectra of DR1 dye-doped PMMA polymer films with extra surface dye before and after plasma modification for different treatment times: (**a**) before plasma treatment (**b**) 300 s (**c**) 420 s (**d**) 720 s (**e**) 1020 s (**f**) 1320 s (**g**) 1620 s plasma treatment.

The DR1 dye-doped PMMA polymer film was immersed in DR1 dye solution (in Ethanol solvent 2 × 10^–3^ M) to adsorb extra dye to its surface in this section of the experiment. Absorption spectra were taken from the sample before and after it was placed in an argon glow discharge plasma reactor for various time intervals. Figure 5 depicts the absorption spectra of the sample at various times of plasma treatment. Figure 6 demonstrates the deconvoluted peaks of each spectrum of Fig. 5 that shows the overlapped peaks related to cis and trans forms of the dye. Table 3 presents the area under each fitted peak related to the population of cis and trans isomers of the dye.

**Table 3:** The amounts of DR1 dye isomeric species and their ratios calculated from fitted double peaks before and after plasma treatment at different time intervals for dye-doped PMMA with extra surface dye.

Samples	S_trans_	S_cis_	S_cis_/S_trans_	S_cis_/S_total_ (%)
Before plasma treatment	141.93	11.73	0.08	7.63
300 s plasma treatment	96.14	28.83	0.29	23.06
420 s plasma treatment	95.34	29.15	0.30	23.41
720 s plasma treatment	90.37	32.40	0.35	26.39
1020 s plasma treatment	83.72	32.97	0.39	28.25
1320 s plasma treatment	80.87	34.96	0.43	30.18
1620 s plasma treatment	76.98	35.32	0.45	31.45

*S*_*trans*_ area under the fitted peak 1, *S*_*cis*_ area under the right fitted peak 2, *S*_*total*_ area under the cumulative fit peak.

### Plasma effect on aging of DR1 dye-loaded PMMA polymer

**Figure 7:**
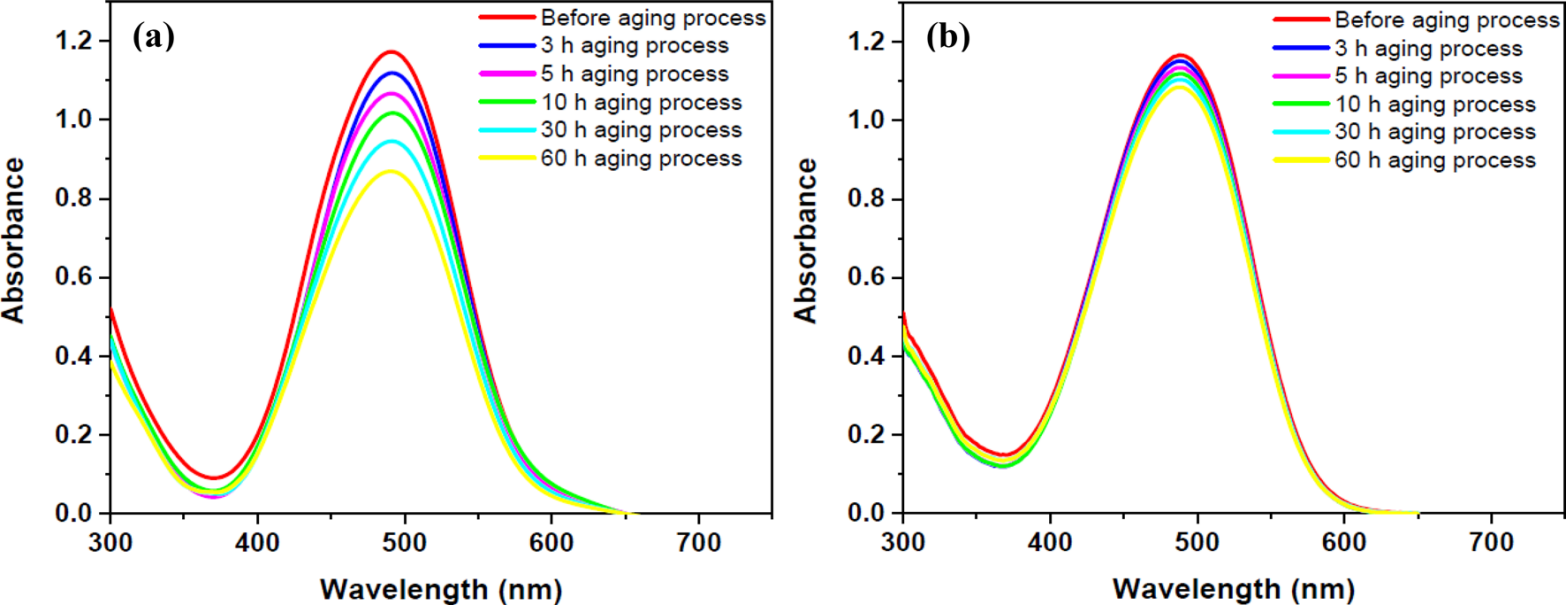
UV–Vis spectra of DR1 dye-loaded PMMA polymer films after different times of aging process: (**a**) untreated dye-loaded film (**b**) plasma modified dye-loaded film for treatment time of 300 s.

The accelerated aging process using intensified conditions measures resistance of substance to heat, UV irradiation, humidity, and other factors in order to obtain results faster than long-term natural aging. The aging effect is an important consideration when estimating dye immobilization on the surface of a material. UV–Vis spectrophotometry was used to investigate the effects of aging process and to estimate dye leaching from the surface of the polymer in this section. Following the preparation of pure PMMA polymer films and their immersion in DR1 dye solution, the samples were placed in a dark environment at a temperature of 10 °C to prevent temporary effects, after they were exposed to argon glow discharge plasma for 300 s. The samples were washed in ethanol before aging process. The accelerated aging process was used to test the degree of dye adhesion to the surface at various time intervals. Finally, UV–Vis spectrophotometry was used to assess dye durability on the polymer film, which was compared to the control sample. The UV–Vis spectra of the untreated and plasma-treated dye-loaded PMMA films (prepared by immersing pure PMMA film in dye solution) after various aging times are shown in Fig. 7 to provide a quantitative description of color fastness. The absorbance spectra of dye-loaded PMMA films examined by UV–Vis spectroscopy show that the dye-loaded PMMA layer treated with plasma has higher dye fastness than similar sample without plasma modification during the various times of aging discoloration process.

## Discussion

The analysis of FT-IR spectra of pristine and plasma-treated pure and dye-doped PMMA samples reveals that plasma has significant impact on some specific peaks. A considerable increase in the intensity of C=O peak at 1732 cm^−1^ is observed that signifies the formation of carbonyl groups on the shallow layer near the surface. Besides, the C=O peak becomes broader due to introduction of some new carbonyl species. The comparative study of FT-IR measurement results indicates that the concentration of C–O bonds changes. Also, there is an obvious decrease in methoxy groups containing OCH_3_ bonds as illustrated in Fig. 1.

**Figure 8:**
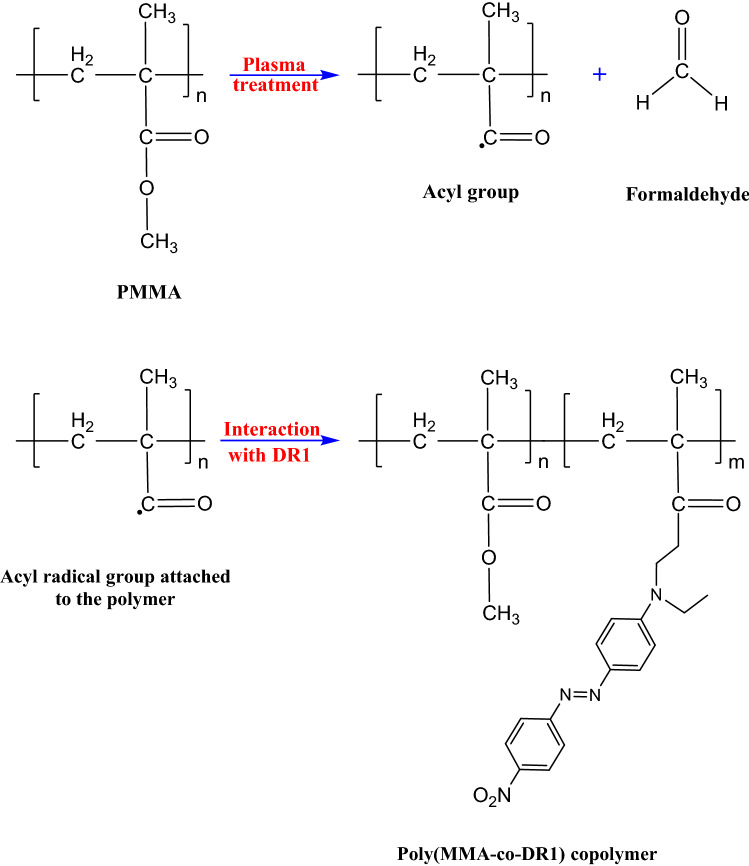
Schematic illustration for chemical changes of PMMA polymer doped with DR1 dye structure due to plasma treatment.

Plasma can create an adequate amount of free radicals or radical sites on the polymer surface through processes such as hydrogen abstraction and homolytic cleavage, resulting in surface activation and the initiation of new reactions^52^. According to the observed results, the following mechanisms are proposed as the effects of plasma on the surface of studied samples:

Some radical structures may form by absorbing ultraviolet radiation in plasma environment and undergo reactions such as hydrogen abstraction and Norrish reactions^53^. In plasma, high-energy particles, in addition to ultraviolet radiation, can greatly enhance the amount of created radicals. Figure 8 shows how pendant groups of the PMMA polymer are cleaved in the place of the C–O bond at the link junction of the methoxy group in the side group of the polymer main chain, resulting in the introduction of acyl radical structures connected to the polymer backbone on the surface of polymer. Breakdown of methoxy group in PMMA structure as a result of C–O bond breakage in the side group of the polymer leads to the formation of formaldehyde and certain radical sites in the polymer structure, which may undergo further reactions. This assertion is consistent with findings from FT-IR spectra, which shows that methoxy groups are reduced and carbonyl groups are generated on the surface. The hydroxyl group in DR1 dye is able to separate from the dye under certain conditions^54^. Through the introduction of certain radicals and composition by plasma, the covalent bond is the fundamental reason for dye immobilization on the polymer film^51^. The dye can form a covalent link with the polymer through produced acyl radicals on the surface of polymer. As a result of the plasma treatment, a layer of poly[(methyl methacrylate)-co-(Disperse Red 1 methacrylate)] (poly(MMA-co-DR1) copolymer forms on the surface of the DR1 dye-doped PMMA polymer. This copolymer is a type of DR1 azo dye copolymer that is created in the form of a surface layer by plasma treatment and is particularly useful in optical researches. This process is a novel and cost-effective method to synthesize this copolymer on the surface homogeneously.

Plasma has the ability to affect substances containing acrylate or methacrylate moieties. This finding is consistent with the literature, which claims that dyes with acrylate or methacrylate functional groups can be immobilized well on polymer surfaces because plasma breaks C–O bonds of acrylate moieties in the special dye structure mentioned in the literature and forms new covalent bonds with the substrate material^51,55^. As a by-product, formaldehyde with the chemical formula of CH_2_O has a polar functional group of C=O. Due to plasma treatment, Van der Waals attractive interactions between this dipolar molecule and the plasma-treated surface of the polymer allow formaldehyde molecules to be adsorbed into the surface. The formed formaldehyde on the PMMA surface can last for a long period. Persistence of some formaldehyde molecules on some specific surfaces is in agreement with the literatures^56,57^. It can act as a self-cleaning surface because of its disinfectant qualities. Also, formaldehyde is a possible intermediary that aids dye adhesion to the polymer surface.

The active surface of plasma-treated sample, which is exposed to air after plasma treatment, may react with the oxygen of ambient air and form oxygen containing species, and some components like low molecular weight oxidized material (LMWOM) are generated on the surface^7,58^. Enhancement of species including oxygen on the surface of polymer such as C=O and C–O bonds plays a crucial role in increasing the concentration of polar functionalities on the surface. Then plasma treatment of PMMA surface can be a satisfactory way to improve surface polarity. Furthermore, the peaks related to C=O bonds connected to carboxyl functional groups are slightly thicker and expand a little towards the area of C–H stretching bonds in FT-IR spectra. The comparison of the FT-IR spectra of the samples before and after plasma treatment reveals that a carboxylic function is created on the surface of the films as a result of subsequent reactions. It is consistent with the findings of Bagiatis et al.^7^. As a result, formic acid is one of the probable species to form on the surface. Because some of the formaldehyde molecules created on the surface by plasma can be oxidized by atmospheric oxygen after contact of the treated samples with ambient air, resulting in the formation of formic acid. The generated carbonyl and carboxyl groups on the surface of the polymer lead to dye immobilization. Because of the substitutions, the carboxylic groups may adhere the dye to the polymer surface, which is consistent with some literatures^59–61^. Plasma can also produce other acyl groups by reacting with the formed formaldehyde, which is highly reactive and can help the dye attach to the polymer through subsequent reactions, and it is in agreement with the literatures^62–64^.

There is a significant decline in the amount of C=C bonds. FT-IR spectra in Fig. 1 show that certain C–H bonds are broken as a result of plasma treatment, as seen by the change in spectra. Plasma treatment of polymer results in substantial dehydrogenation and deprotonation reactions^65^. Another impact by noble gas plasma may be the formation of carbon–carbon bonds as a result of hydrogen desorption^8^. At the surface of polymers, argon gas plasma can cause a reaction known as "CASING" (Cross-linking by Activated Species in INert Gases)^51^. Separation of hydrogen atoms by Ar ions can result in the formation of reactive structures on the surface of the polymer for subsequent processes like cross-linking and polymer branching. Cross-linking processes may occur in the result of etching and sputtering of the structures with C=C bonds on the surface leading to reduction of the concentration of C=C bonds and dehydrogenation of the polymeric surface, and introduce additional C–C bonds, resulting in longer polymer chains.

The changes of UV–Vis spectra of Fig. 2 are justified for two reasons: one is due to dye conformation modification, and the other is due to etching and sputtering of surface components by heavy and energetic particles in plasma. These two alternatives will be addressed in the following.

The conformational changes of the dye doped in the polymer film by plasma treatment may be assessed by UV–Vis spectroscopy. There are two appearing peaks for each conformation in the UV–Vis spectra of DR1 dye assigned to π → π* and n → π* transitions. In the trans isomers, the intensity of absorption band of π → π* transition is higher than the cis species^42^. It is necessary to mention, in DR1 dye as a pseudo-stilbene type dyes, the two peaks for each conformation in the UV–Vis absorption spectrum are overlapped^66^. The UV–Vis absorption spectra were fitted by two Gaussian bands using the Origin 2018 software to produce fitted peaks of cis and trans isomers and the area under the deconvoluted curves to understand the amount of changes in the population of cis and trans isomers. The area under the deconvoluted curves from the UV–Vis absorption spectra of the samples was used to calculate the cis to trans isomers ratio. Figure 3 indicates the cumulative and deconvoluted curves obtained from the absorption spectra of the dye-doped polymer film before and after plasma treatment in various times.

As seen in Table 1, plasma increases the population of cis forms by transferring energy to trans species. Plasma induces reorientation of the polymeric components on the surface around the dye molecules, and makes it harder for the dye to return from the cis to the trans form and stabilizes the dye in the cis state. Substitutions and changes in the structure of the surface components and the presence of different products created on the surface by plasma are the other factors affecting the stabilization of the dye in the cis mode and resulting in changes in absorption wavelength of both peaks of conformational species.

Table 1 summarizes the calculated areas under the fitted curves, which illustrates the changes in the population of cis and trans isomers before and after plasma treatment at various time intervals. After a month, there was no change in the transfer of the cis states to the trans forms of the dye in the samples.

According to Table 2 information, the R_a_ value increased significantly after plasma treatment. Also, AFM results show enhancement of R_z_ and R_q_ roughness parameters. In a plasma environment with DC bias, irradiation of heavy and energetic Ar ions generate scratches on the surface of the sample, and plasma builds nanostructures on the surface by using processes like etching and sputtering. During plasma treatment, certain grooves and porosities are formed on the surface of the polymer, with groove thicknesses ranging from 400 to 600 nm. Furthermore, the freshly created radicals and active surface of PMMA can react with other dangling chemicals, causing certain molecules to cling to the polymer and form bumps. As can be seen in Fig. 4, argon plasma can induce worm-like chain structure network on the surface of polymer that increases the surface roughness. The surface topography of the material and the size of the contact surface have an impact on adhesion. Increased surface roughness enhances the contact surface of dye molecules with the polymer macromolecules, which strengthens dye adherence on the surface.

Figure 6 shows the fitted and cumulative lines of the spectra of the DR1 dye-doped PMMA polymer film with extra surface dye before and after various times of plasma treatment. Table 3 summarizes the information obtained from fitting diagrams. The concentration of dye molecules on the surface is higher in this section than the previous one. When the results of this section in Table 3, are compared to the results of the similar tests for dye-doped polymer film without additional surficial dye in Table 1, it can be concluded that plasma has a greater effects on the surface of the polymer and conformation changes of dye molecules present on the surface than dye molecules in the bulk of sample. Because in a plasma environment, photons and energetic particles can collide with the surface and have a greater impact on the dye molecules on the surface of polymer. On the other hand, the dye molecules on the surface of the polymer have more freedom for conformational changes. Plasma creates special physical and chemical structures and particular substituents on the surface that allow the cis form of dye to maintain its stability.

Chemical changes, oxidation, eliminating weak connections, and reorientation all contribute to the aging process of dye-loaded surface of polymeric material^67,68^. In addition, photodegradation caused by UV radiation in the presence of heat and humidity contributes significantly to the accelerated aging of the sample. The results show that plasma modification causes less surface discoloration during the aging process due to the creation of strong dye connections to the polymer surface, and the covalent bonds produced by the plasma treatment, play an important role in dye immobilization as the strongest links. Of course, the other induced phenomena, such as surface pores and changes of conformation of dye molecules aid dye adhesion to the polymer surface.

## Conclusions

The effects of argon DC glow discharge plasma were investigated on the pure and DR1 dye-loaded PMMA films. During plasma treatment, charged and energetic particles penetrates the surface of the sample and affect some bonds due to an electrical potential gradient. Furthermore, energetic particles and photons in the plasma environment attack the dye-loaded PMMA surface, altering some of its properties. Plasma treatment improves dye immobilization on the surface of polymer through activation of the surface by producing radicals and covalent bonds between the dye and the polymer. This technique is a cost-effective method for coloring the material due to the disusing of dye in the bulk of polymer leading to low consumption of dye. Plasma can cause phenomena like dipole–dipole interactions and hydrogen bonding on the surface of polymers because of formation of polar terminal groups, such as carbonyl and carboxyl. The oxygen-containing groups can adsorb dye molecules with hydrogen atoms and are considered donors in hydrogen bonding enhancing the adhesion of the dye to the surface of the polymer. A layer of (poly(MMA-co-DR1)) copolymer can be synthesized homogeneously on the surface using this method, which involves plasma treatment of DR1 dye-loaded PMMA polymer. The surface roughness has significant effect on increasing the dye fastness on the surface by enhancement of the contact surface area for dye molecules. The population of cis isomers of the dye grows under the exposure of plasma at a proper rate. By increasing the amount of cis isomers of dye on the surface of polymer, covalent bonding is more likely because of the higher energy state of cis species, and plasma helps the dye adhere better to the surface. DC plasma forms new structures on the surface and reoriente some of the components of the surface in the direction of the field. In addition to this factor, the stability of the cis form is affected by increasing the surface pores and producing new structures and substituents. All of these features increase the functional capacity of such materials, and allow them to be used in optical applications and data storage. Plasma treatment provides longer dye retention on the surface of polymer for use in holographic and halochromic applications. Also, the increased stable cis species of the dye on the surface of polymer reduces polymer degradation due to more radiation absorption in UV range.

## Materials and methods

### Materials

PMMA polymer powder with an average molecular weight of 120,000 and a density of 1.88 g/ml at 25 °C was purchased from Sigma-Aldrich for use in this work. Merck provided DR1 dye, dichloromethane, and ethanol as solvents, while the water used in this experiment was distilled water.

### Characterization instruments

Vertex 70 scan spectrophotometer was used to obtain absorbance FT-IR spectra in the wavenumber range of 400–4000 cm^−1^. The fully digital FT-IR spectrophotometer features technology based on parallel running dual-channel delta sigma with 24-bit dynamic range. The accuracy of this spectrophotometric tool is 3 cm^−1^. A double beam Shimadzu UV-2450 scan UV–Visible spectrophotometer was used to measure UV–Vis spectra in the wavelength range of 200–900 nm with medium scanning speed. This spectroscopic instrument were combined with a cell temperature controller with an accuracy of ± 0.1 °C. The surface topographical modification was seen using an atomic force microscope (AFM) of the Nanosurf Mobile-S type with 2 controller and Si_3_N_4_ needle. The surface morphology of the samples was determined by dynamic force operating mode with vibrational frequency of 170 kHz.

### Aging test

To identify and compare the degree of dye attachment to the surface of polymer films, an accelerated aging procedure was chosen. The dye-loaded PMMA films, both untreated and plasma-treated, were exposed to ultraviolet radiation in a damp and warm atmosphere. The samples were kept in a chamber with quartz walls positioned at identical distances and angles from a 500 W mercury-vapor UV source under ambient air pressure at 55 °C and 87% humidity. Distilled water was used to maintain the humidity of the chamber. Variation in the spectra obtained by UV–Vis spectrophotometer was used to estimate the color changes caused by the aging process of the PMMA films.

### Sample preparation

The polymer solution was made by dissolving PMMA powder in dichloromethane solvent at a concentration of 0.6 W/W% at room temperature. The PMMA films were casted by spin-coating method from the solution on quartz slides. Three batches of polymer films were made: the first batch consisted of pure polymer films that were used to determine the chemical changes caused by plasma and study dye stabilization by plasma on the surface of pure film after dipping in dye solution and plasma through aging process. The second batch of polymer films was made from a PMMA polymer solution that had been doped with DR1 dye at a concentration of 2 W/W%. The third category included pure and DR1 dye-doped PMMA films that were immersed in a DR1 dye solution in ethanol solvent at a concentration of 15 mg/ml for 120 s to adsorb extra dye homogeneously on the surface of polymer, which were approximate optimized dye concentrations and dipping times for high dye loading on the surface obtained by trial and error. The samples were dried in an oven at 35 °C. The samples were then placed in a plasma environment. In several circumstances, the effects of plasma on the samples were compared to the untreated control samples. All of the tests were carried out twice.

### Plasma instrument

A Pyrex cylindrical tube with a gap length of 500 mm was used as the DC glow discharge plasma device chamber in this study. The Pyrex glass chamber was connected and sealed by two aluminum parallel electrodes at the ends. The samples were placed in the positive column zone of plasma. The plasma chamber was filled with pure argon gas (with a purity of 99.99%). Before turning on the discharge, the chamber was connected to an Alcatel rotary vacuum pump to be evacuated of air molecules and purged with the target gas. Then, at a work pressure of argon gas at 2 × 10^–1^ Torr, Plasma was created at the distance between two electrodes after the electrodes were connected to a power supply. To accelerate charged particles inside the plasma reactor, the applied DC voltage between two electrodes was kept at 1.2 kV with a discharge current of 0.15 A. The plasma had a power density of 720 mW/cm^3^. After plasma treatment, ambient air was introduced into the chamber, bringing the pressure to atmospheric levels. The samples were left unused in a dark place for up to 16 h after plasma treatment to reduce some temporary and weak effects.

### Statistical analysis

The UV–Vis absorption spectra were fitted by two Gaussian bands using the Origin software (OriginPro 2018 (64-bit) SR1; b9.5.1.195), (http://www.OriginLab.com) to produce fitted peaks of cis and trans isomers and the area under the deconvoluted curves. The area under each fitted peak expresses scale amount of the population of isomers species. The area under the deconvoluted curves from the UV–Vis absorption spectra of the samples was used to calculate the cis to trans isomers ratio. Also, smoothing of raw data was accomplished by this software using the Savitzky-Golay method with 32 points of window, and with polynomial order 2.

## Supplementary Information

Supplementary Figures.

## Data Availability

The datasets obtained during the current study are available from the corresponding author on reasonable request.
