# Supplementary material for: Glow discharge plasma stabilization of azo dye on PMMA polymer
Source: Sci Rep. 2022 Nov 1;12:18358. doi: 10.1038/s41598-022-21855-4 (PMC9626643; doi:10.1038/s41598-022-21855-4)
Supplement: Supplementary file 1 — Supplementary Figures. [file 41598_2022_21855_MOESM1_ESM.pdf]

Supplementary information

Glow Discharge Plasma Stabilization of Azo Dye on PMMA Polymer

Mohammad Reza Yari[1,*,+], Mohammad Sadegh Zakerhamidi[2,3,4], and Hamid Ghomi[1,*,+]

[1] Laser and Plasma Research Institute, Shahid Beheshti University, Tehran, Iran.

[2] Faculty of Physics, University of Tabriz, Tabriz, Iran.

[3] Research Institute for Applied Physics and Astronomy, University of Tabriz, Tabriz, Iran.

[4] Photonics Center of Excellence, University of Tabriz, Tabriz, Iran.

* Corresponding authors

Mohammad Reza Yari, E-mail: M_Yari@sbu.ac.ir

Hamid Ghomi, E-mail: h-gmdashty@sbu.ac.ir

[+] These authors contributed equally to this work.

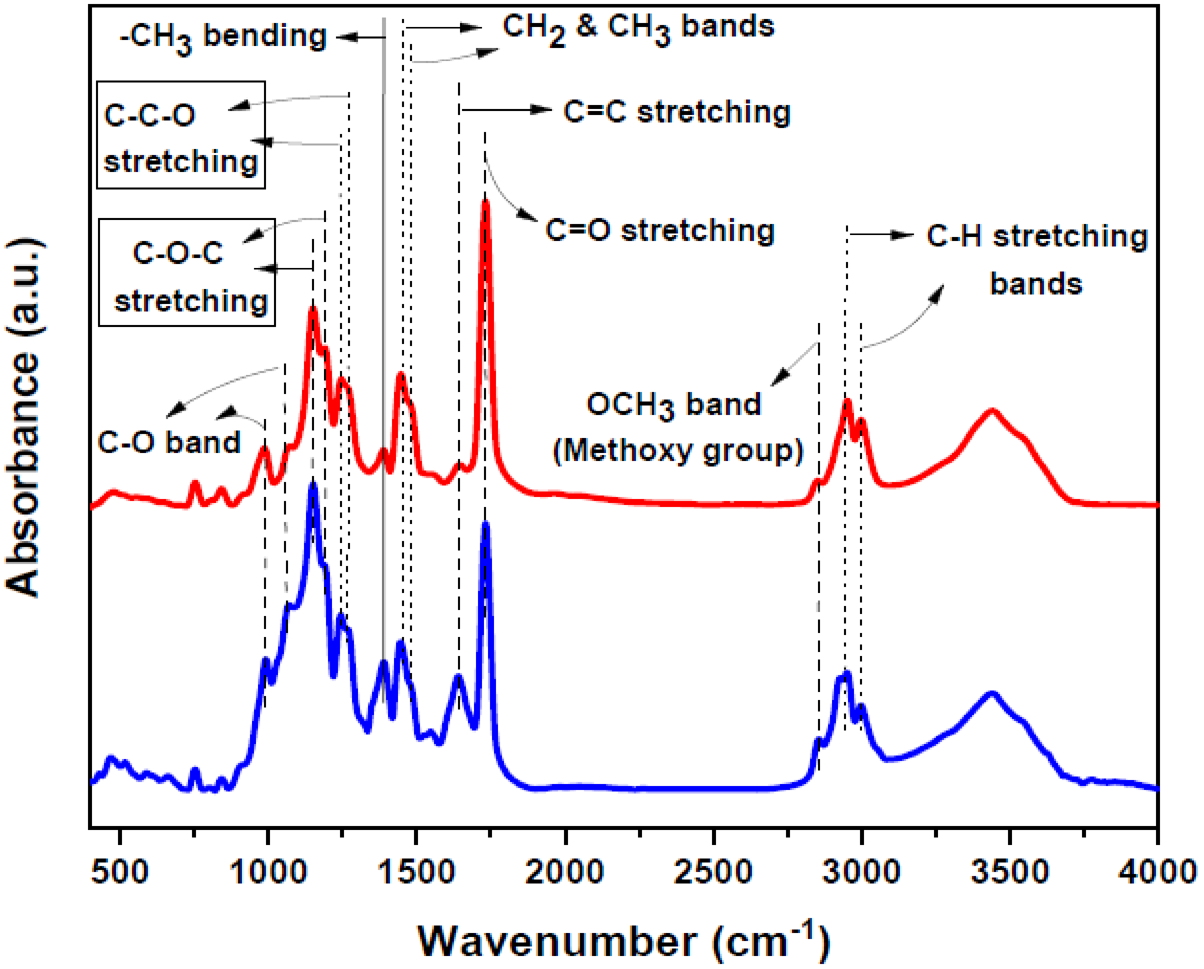

Figure S1. The detailed peak information of FT-IR spectra of PMMA polymer (blue lines) untreated sample (red lines) argon plasma-treated sample for 300 s

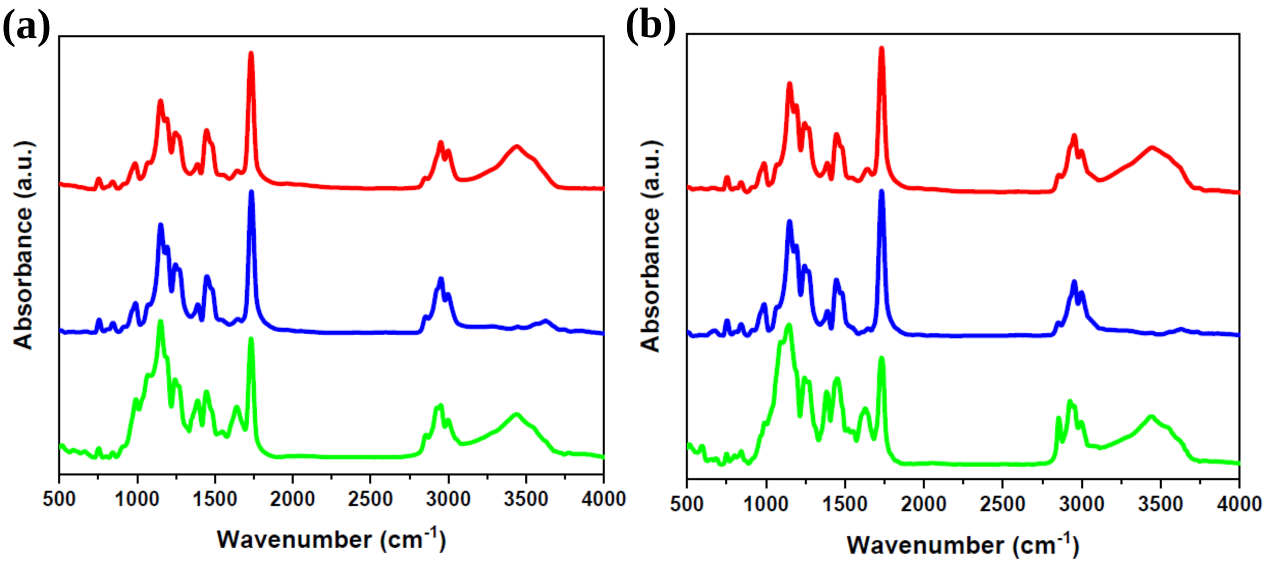

Figure S2. FT-IR spectra of (a) pure PMMA film (b) DR1 dye-doped PMMA polymer film (green lines) untreated samples (blue lines) argon plasma-treated samples for 150 s (red lines) argon plasma-treated samples for 300 s

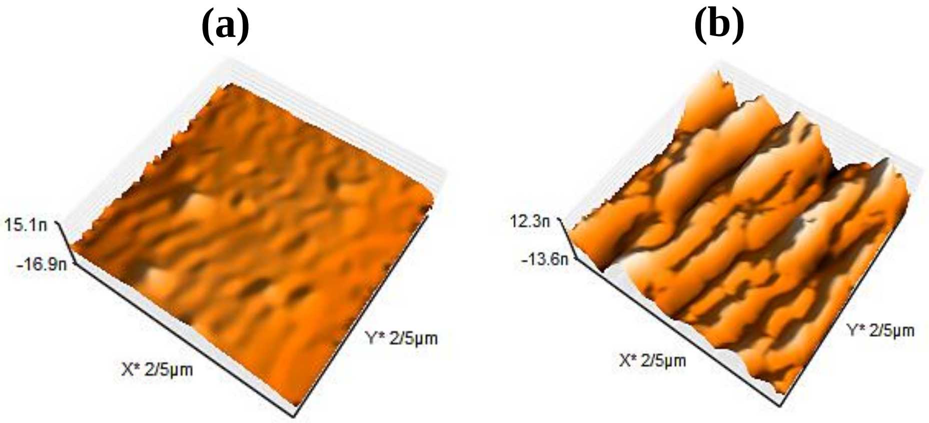

Figure S3. AFM images of (a) untreated PMMA surface (b) argon plasma treated PMMA surface for 150 seconds modification
